# Increased risk of dementia following herpes zoster ophthalmicus

**DOI:** 10.1371/journal.pone.0188490

**Published:** 2017-11-22

**Authors:** Ming-Chieh Tsai, Wan-Ling Cheng, Jau-Jiuan Sheu, Chung-Chien Huang, Ben-Chang Shia, Li-Ting Kao, Herng-Ching Lin

**Affiliations:** 1 Department of Internal Medicine, Hsinchu Cathay General Hospital, Hsinchiu, Taiwan; 2 School of Health Care Administration, Taipei Medical University, Taipei, Taiwan; 3 Department of Infectious Disease, Hsinchu Cathay General Hospital, Hsinchiu, Taiwan; 4 Department of Neurology, Taipei Medical University Hospital, Taipei, Taiwan; 5 Department of Neurology, School of Medicine, College of Medicine, Taipei Medical University, Taipei, Taiwan; 6 Big Data Research Center, Taipei Medical University, Taipei, Taiwan; 7 Graduate Institute of Life Science, National Defense Medical Center, Taipei, Taiwan; University of California Riverside, UNITED STATES

## Abstract

This retrospective cohort study aimed to examine the relationship between herpes zoster ophthalmicus (HZO) and the subsequent risk of dementia using a population-based database. We retrieved the study sample from the Taiwan Longitudinal Health Insurance Database 2005. The study group included 846 patients with HZO, and the comparison group included 2538 patients without HZO. Each patient was individually followed for a 5-year period to identify those patients who subsequently received a diagnosis of dementia. We performed a Cox proportional hazards regression to calculate the hazard ratios (HRs) along with 95% confidence intervals (CIs) for dementia during the follow-up period between patients with HZO and comparison patients. The respective incidence rates of dementia per 1000 person-years were 10.15 (95% CI: 7.22~13.87) and 3.61 (95% CI: 2.61~4.89) for patients with HZO and comparison patients. The Cox proportional analysis showed that the crude HR of dementia during the 5-year follow-up period was 2.83 (95% CI: 1.83–4.37) for patients with HZO than comparison patients. After adjusting for patients’ characteristics and comorbidities, HZO patients were still at a 2.97-fold greater risk than comparison patients for developing dementia. Furthermore, we found that of sampled male patients, the crude HR of dementia for patients with HZO was as high as 3.35 (95% CI = 1.79–6.28) compared to comparison patients. This study demonstrated an association between HZO and dementia. Clinicians must be alert to suspect dementia in patients with cognitive impairment who had prior HZO.

## Introduction

Dementia is a clinical syndrome caused by damage to cerebral nerve cells and is characterized by a decline in mental ability severe enough to interfere with daily life [1]. Epidemiological studies have identified many common cardiovascular risk factors for dementia, such as age, diabetes mellitus, smoking, hypercholesterolemia, physical inactivity, increased fat intake, metabolic syndrome, and hypertension. Previous studies also reported evidence of considerable cerebrovascular pathology in Alzheimer’s disease (AD). The toxic effects of vascular factors on the brain microvasculature may cause cerebral hypoperfusion and ischemia resulting in angiogenesis, which synergizes with β-amyloid to produce AD [1–9].

Varicella zoster virus (VZV) is the only human virus that can replicate in cerebral arteries and produce vasculopathy, mainly in the elderly and immunocompromised patients [10,11]. The virus spreads transaxonally to cerebral arteries from the trigeminal nerve, particularly from the ophthalmic branch of trigeminal afferent fibers and induces further vascular inflammation and thrombosis that can subsequently injure brain cells [12–14]. Previous reports established that VZV vasculopathy potentially results in transient ischemic attacks, ischemic or hemorrhagic stroke, aneurysms, venous sinus thromboses, and arterial dissection [11,13]. Thus, an increased risk of dementia following VZV vasculopathy or cerebral arteritis with subsequent brain cells damage is a reasonable hypothesis.

Furthermore, previous studies found that among all types of herpes zoster, patients with herpes zoster ophthalmicus (HZO) had a more-marked risk of stroke and vasculopathy than other types of herpes zoster [11–13,15–17]. Several studies also reported that a stroke increases the risk of dementia [18–20]. HZO may therefore be considered a common risk factor in the pathogenesis of dementia and VZV vasculopathy. However, the association between HZO and dementia remains unclear. This retrospective cohort study aimed to examine the relationship between HZO and the subsequent risk of dementia using a population-based database.

## Methods

### Database

We retrieved the study sample for this retrospective cohort study from the Taiwan Longitudinal Health Insurance Database 2005 (LHID2005). The LHID2005 includes medical claims of healthcare services for 1,000,000 enrollees who were systematically and randomly selected from all enrollees (*n* = 25.68 million) listed in the 2005 Registry of Beneficiaries under the Taiwan’s National Health Insurance (NHI) program. The LHID2005 allows researchers in Taiwan to longitudinally follow-up medical services for these 1,000,000 enrollees since initiation of the NHI in 1995.

This study was approved by the joint institutional review board (JIRB) of Taipei Medical University (TMU-JIRB 201702027).

### Study sample

This study was designed to include a study group and a comparison group. We selected the study group by first identifying 1389 patients who had received a first-time principal diagnosis code of HZO (ICD-9-CM codes 053.2, 053.20, 053.21, 053.22, or 053.29) in clinics or in hospitals between January 1, 2001 and December 31, 2008. We excluded 437 sampled patients who were aged <40 years because of the very low incidence of dementia in this age group. We then assigned the date of receiving their first-time diagnosis of HZO as the index date. We further excluded 70 sampled patients who had a history of major psychosis or a substance-related disorder (ICD-9-CM codes 291~299 or 303~305) prior to the index date. We also excluded 36 sampled patients who had a history of dementia (ICD-9-CM codes 290.0~290.4, 294.1, 331.0~331.2, or 331.82) before the index date. As a result, 846 patients with HZO were included in the study group.

As to the selection of the comparison group, we likewise selected comparison patients from the remaining enrollees in the registry of beneficiaries of the LHID2005. We used propensity score matching to select 2538 comparison patients (three for every study patient with HZO). The selected variables including age, sex, monthly income, geographic location, urbanization level, the year of the index date, hypertension, diabetes, hyperlipidemia, coronary heart disease, and stroke were entered into a multivariable logistic regression model as predictors to calculate the expected probability of receiving a diagnosis of HZO for each patient. In this study, the year of the index date was the year in which patients had received their first-time diagnosis of HZO. Comparison patients were selected by matching them to a given patient with HZO simply on their utilization of medical services in the same index year of that particular case. We further assigned the first use of medical services in the index year as the index date for the comparison group. We assured that all selected comparison patients had not received a diagnosis of herpes zoster within 1 year before the index date. Furthermore, we assured that none of the selected comparison patients had a history of major psychosis, substance-related disorder, or dementia prior to their index date.

There were 3384 sampled patients including 846 study patients and 2538 comparison patients. Each patient was individually followed for a 5-year period starting from their index date to identify those patients who subsequently received a diagnosis of dementia. To avoid issues associated with the diagnostic validity of the administrative dataset, this study only include patients diagnosed with dementias by a certified neurologist. In addition, dementia cases only included those patients who had received two or more dementia diagnoses.

### Statistical analysis

All statistical analyses were performed with SAS statistical software and the significance level was set to 0.05 in this study. We performed a Cox proportional hazards regression to calculate hazard ratios (HRs) along with 95% confidence intervals (CIs) for dementia during the 5-year follow-up period between patients with HZO and comparison patients. The proportional hazards assumption was tested, and we found that survival curves for both strata (patients with HZO and comparison patients) had hazard functions that were proportional over time. In this study, we also censored patients who died or were lost to follow-up during the 5-year follow-up period (155 from the study cohort (18.3% of patients with HZO) and 428 from the comparison cohort (16.9% of comparison subjects).

## Results

Of the study sample, the mean age at the index date was 61.6 years with a standard deviation of 13.1 years; mean ages were 62.2 (±12.5) years for patients with HZO and 61.4 (±13.3) years for comparison patients (*p* = 0.112). Table 1 shows that there was no significant difference in sex, age, urbanization level and geographic location of patients’ residences, monthly income, hypertension, diabetes, hyperlipidemia, coronary heart disease, and stroke between patients with HZO and comparison patients.

**Table 1:** Demographic characteristics of sampled patients in Taiwan, stratified by the presence of herpes zoster ophthalmicus (HZO) (N = 3384).

Variable	Patients with HZO *N* = 846		Comparison patients *N* = 2538		*p* value
	Total no.	Column %	Total no.	Column %	
Male	420	49.7	1318	51.9	0.249
Age (years), mean (SD)	62.2±12.5		61.4±13.3		0.112
Geographic region					0.163
Northern	304	35.9	920	36.3	
Central	151	17.9	420	16.6	
Southern	389	46.0	1173	46.2	
Eastern	2	0.2	25	1.0	
Urbanization level					0.965
1 (most urbanized)	234	27.7	707	27.9	
2	189	22.3	593	23.4	
3	196	23.2	584	23.0	
4	102	12.1	294	11.6	
5 (least urbanized)	125	14.8	360	14.2	
Monthly income ^a^					0.762
NT$0~15,840	345	40.8	1010	39.8	
NT$15,841~25,000	340	40.2	1017	40.1	
≥NT$25,001	161	19.0	511	20.1	
Hypertension	458	54.1	1321	52.1	0.292
Diabetes	210	24.8	569	22.4	0.150
Hyperlipidemia	312	36.9	910	35.9	0.591
Stroke	162	19.2	446	17.6	0.301
Coronary heart disease	234	27.7	657	25.9	0.311

^a^ The average exchange rate for 2010 was US$1.00≈New Taiwan (NT)$30; SD = standard deviation.

Incidence rates of dementia within the 5-year period following their index dates are presented in Table 2. The incidence rate of dementia was 5.24 (95% CI: 4.61~6.51) per 1000 person-years among all sampled patients; respective incidence rates of dementia per 1000 person-years were 10.15 (95% CI: 7.22~13.87) and 3.61 (95% CI: 2.61~4.89) for patients with HZO and comparison patients. Furthermore, the log-rank test indicated that there was a statistically significant difference in 5-year dementia-free accumulated survival rates between patients with HZO and comparison patients (*p*<0.001).

**Table 2:** Crude hazard ratios (HRs) and 95% confidence intervals (CIs) for dementia among sampled patients during the 5-year follow-up period.

Presence of a dementia diagnosis	Total sample *N* = 3384 *n*, %		Patients with herpes zoster ophthalmicus *N* = 846 *n*, %		Comparison patients *N* = 2538 *n*, %	
Five-year follow-up period						
Yes	81	2.39	39	4.61	42	1.65
Incidence rate per 1000 person-years (95% CI)	5.24 (4.61–6.51)		10.15 (7.22–13.87)		3.61 (2.61–4.89)	
Crude HR (95% CI)	-		2.83*** (1.83–4.37)		1.00	

Notes: HRs were calculated by a Cox proportional hazard regression.

*** p<*0*.*001*.

Table 2 also analyzes HRs for dementia during the 5-year follow-up period for patients with HZO and comparison patients. The Cox proportional analysis showed that the crude HR of dementia during the 5-year follow-up period was 2.83 (95% CI: 1.83~4.37) for patients with HZO compared to comparison patients, after censoring those who died and were lost to follow-up during the follow-up period. Additionally, the covariate-adjusted HR of dementia was 2.97 (95% CI: 1.90–4.67) for patients with HZO compared to comparison patients after adjusting for sex, age, urbanization level and geographic location of patients’ residences, monthly income, hypertension, diabetes, hyperlipidemia, coronary heart disease, and stroke (Table 3).

**Table 3:** Covariate-adjusted hazard ratios (HRs) for the occurrence of dementia during the 5-year follow-up period among patients with herpes zoster ophthalmicus.

Variable	Dementia incidence		
	Hazard ratio	95% CI	*p* value
Herpes zoster ophthalmicus	2.97	1.90–4.67	<0.001
Age	1.12	1.09–1.15	<0.001
Sex	0.86	0.55–1.36	0.522
Geographic region			
Northern	1.00		
Central	0.36	0.15–0.83	0.017
Southern	0.90	0.54–1.50	0.692
Eastern	3.82	0.50–29.25	0.197
Monthly income			
NT$0~15,840	1.00		
NT$15,841~25,000	0.59	0.32–1.10	0.095
≥NT$25,001	1.39	0.51–3.76	0.521
Urbanization level			
1 (most urbanized)	1.00		
2	0.48	0.23–1.01	0.052
3	0.85	0.46–1.59	0.618
4	1.19	0.54–2.61	0.673
5 (least urbanized)	0.86	0.39–1.90	0.713
Hypertension	2.09	1.01–4.34	0.048
Diabetes	1.48	0.92–2.36	0.103
Hyperlipidemia	1.59	1.00–2.53	0.050
Coronary heart disease	0.72	0.45–1.16	0.172
Stroke	1.70	1.07–2.71	0.024

Notes: All hazard ratios were derived from the same Cox regression model and adjusted for all other variables.

Furthermore, the issue of surveillance bias might have occurred in this study, because many researchers consider that patients with HZO have a greater tendency to receive neurological examinations, and therefore more dementia cases would be detected. We performed a sensitivity analyses by excluding patients who received a diagnosis of dementia within 2 months after their index date (*n* = 4) in order to address this concern. We found that the results remained the same (crude HR = 2.82, 95% CI = 1.80–4.41) (data not shown). The potential surveillance bias thus likely did not compromise the relationship between HZO and dementia reported in this study.

Table 4 further analyzes HRs of dementia between patients with HZO and comparison patients by sex. We found that of the sampled male patients, the crude HR of dementia for patients with HZO was as high as 3.35 (95% CI = 1.79–6.28) compared to comparison patients. In addition, the covariate-adjusted HR of dementia for patients with HZO was 3.41 (95% CI = 1.78–6.53) compared to comparison patients.

**Table 4:** Crude and covariate-adjusted hazard ratios (HRs) and 95% confidence intervals (CIs) for dementia during the 5-year follow-up period by sex.

Presence of a dementia diagnosis	Sex							
	Males				Females			
	Patients with herpes zoster ophthalmicus (*N* = 420) *n*, %		Comparison patients (*N* = 1318) *n*, %		Patients with herpes zoster ophthalmicus (*N* = 426) *n*, %		Comparison patients (*N* = 1220) *n*, %	
Five-year follow-up period								
Yes	20	4.76	19	1.44	19	4.46	23	1.89
Incidence rate per 1000 person-years (95% CI)	10.50 (6.41–16.21)		3.14 (1.89–4.90)		9.80 (5.90–15.30)		4.13 (2.62–6.20)	
Crude HR (95% CI)	3.35*** (1.79–6.28)		1.00		2.40** (1.31–4.41)		1.00	
Adjusted HR^a^ (95% CI)	3.41*** (1.78–6.53)		1.00		2.98** (1.54–5.77)		1.00	

Notes: HRs were calculated by a Cox proportional hazard regression.

*** p<0.001.

** p<0.01.

^a^ Adjusted for age, monthly income, geographical region, urbanization level, hypertension, diabetes, hyperlipidemia, coronary heart disease, and stroke.

## Discussion

This retrospective cohort study showed that 4.61% of the patients with HZO subsequently received a diagnosis of dementia during the 5-year follow-up period compared to 1.65% of patients without HZO. The Cox proportional hazard regression analysis showed that HZO was significantly associated with an increased risk of subsequent dementia (crude HR = 2.83, 95% CI = 1.83–4.37). The high incidence of dementia among HZO patients may be explained by VZV vasculopathy with damage to cerebral neural cells due to cerebral arterial inflammatory and thrombotic responses, which may be a factor leading to dementia [10–16]. Previous studies demonstrated that patients with HZO had higher risks of VZV vasculopathy and stroke than did patients with other types of herpes zoster [15–17]. Studies also mentioned increases in dementia after a stroke, ranging 3%~19% in variable follow-up periods (from 3 months to 10 years) [18–20]. In addition, psychological stress, aging, a poor social support environment, and adverse life events may contribute to both VZV reactivation and dementia [1–3, 21–23].

Some studies suggested that gender is associated with dementia, but their conclusions which only focused on vascular dementia do not permit unequivocal conclusions [3, 5, 24–25]. In a large population-based prospective cohort study, Ruitenberg et al. reported that the incidence of vascular dementia was higher for men than women in all age groups [26]. In our study, we found that the magnitude of dementia risk following HZO was about 1.4 times as high for males compared to females (crude HR = 3.35 vs. 2.40). The mechanism remains unclear, but it may imply that cerebrovascular events play an important role in HZO with dementia. Previous studies reported that stroke is more common in men, which may be explained by the protective effect of estrogen in premenopausal females and the higher risk of cardiovascular diseases in males [27–29]. Förster et al. reported that diseases of large and small vessels are major causes of male stroke compared to cardioembolism in female stroke victims [29]. This parallels VZV vasculopathy following trigeminal-distribution zoster involving large and small arteries ultimately leading to cerebral infarction, which suggests that males with HZO are at a higher risk of stroke and subsequent dementia than females [11].

This study has several strengths. First, a population-based dataset with a large sample size was used to explore the relationship between HZO and dementia during the study period. The large sample size afforded a considerable statistical advantage in detecting real differences between the two cohorts. Second, the diagnosis of HZO and dementia by certified neurologists, infection specialists, and dermatologists has very high validity in Taiwan.

Nevertheless, the results of this study need to be seen in the light of several limitations. The first limitation is that the LHID 2005 provides no information on the history of smoking, body mass index, education, or alcohol consumption, which are considered to be risk factors and may influence the pathogenesis of dementia [3, 4]. This limitation may affect the actual association between HZO and following dementia and may make the relevant analyses incomplete. Second, the absence of a skin rash is well known in VZO- and VZV-related neurological diseases, and because physicians may find it difficult to diagnose such cases, they were not included in our study [30]. Finally, we did not compare the effect of antiviral treatment for HZO with subsequent dementia. However, one study by Lin et al. reported that there was no significant difference in the rate of stroke development between patients who had received systemic antiviral treatment and those who had not [16].

Despite the aforementioned limitations, this study demonstrated a positive association between HZO and dementia. In patients with HZO, VZV vasculopathy may contribute to some degree to the pathogenesis of dementia. Therefore, clinicians must be alert to suspect dementia in patients with cognitive impairment who had prior HZO.
